# Draft Genome Sequence of *Herpetosiphon geysericola* GC-42, a Nonphototrophic Member of the *Chloroflexi* Class *Chloroflexia*

**DOI:** 10.1128/genomeA.01352-15

**Published:** 2015-11-19

**Authors:** Lewis M. Ward, James Hemp, Laura A. Pace, Woodward W. Fischer

**Affiliations:** a Division of Geological and Planetary Sciences, California Institute of Technology, Pasadena, California, USA; b Department of Medicine, University of California San Diego, La Jolla, California, USA

## Abstract

We report here the draft genome sequence of *Herpetosiphon geysericola* GC-42*,* a predatory nonphototrophic member of the class *Chloroflexia* in the phylum *Chloroflexi*. This genome provides insight into the evolution of phototrophy and aerobic respiration within the *Chloroflexi*.

## GENOME ANNOUNCEMENT

The majority of cultured members of the bacterial phylum *Chloroflexi* belong to the class *Chloroflexia*, which are prominently anoxygenic phototrophs (1). However, the most basal *Chloroflexia*, members of the orders *Herpetosiphonales* and *Kallotenuales*, are nonphototrophic (2). This makes these clades of central importance to understanding the evolution of phototrophy within the *Chloroflexi*. The *Herpetosiphonales* order currently contains only two species, *Herpetosiphon geysericola* and *Herpetosiphon aurantiacus*, which are pigmented filamentous organoheterotrophs that exhibit gliding motility (3). In culture, *Herpetosiphon* strains are obligate aerobes that prefer microaerobic conditions (3). The physiology and ecology of these organisms are not well understood, but it has been suggested that members of the *Herpetosiphon* genus might be capable of predation via a “wolf pack” strategy (4). *H. geysericola* was isolated from a biofilm at a hot spring in Baja California, Mexico (5).

The genome of *H. geysericola* GC-42 (DSM 7119) was sequenced as part of a project to expand the phylogenetic breadth of *Chloroflexi* genomes. Genome sequencing was performed at SeqMatic using the Illumina MiSeq sequencing platform. SPAdes 3.1.1 (6) was used to assemble the genome. The genome was screened for contaminants based on sequence coverage, G+C composition, and BLAST hits of conserved single-copy genes. Genome annotation was performed using the NCBI Prokaryotic Genome Annotation Pipeline. The draft genome is 6.24 Mb in size, assembled into 46 contigs. It contains 5,335 genes, 4,688 coding sequences (CDSs), 2 16S RNAs, 47 tRNAs, and 6 clustered regularly interspaced short palindromic repeat (CRISPR) arrays. It is estimated to be ~99% (111/111) complete based on conserved single-copy genes.

Analysis of the *H. geysericola* genome revealed genes for a branched aerobic respiratory chain, including two different complex Is (NADH dehydrogenase), complex II (succinate dehydrogenase), complex III (cytochrome *bc* complex), an A-family heme-copper oxygen reductase, and a quinol *bd* oxidase. No genes for phototrophy were found. In addition, no genes associated with the 3-hydroxypropionate cycle, a CO_2_ fixation pathway present in photosynthetic members of *Chloroflexi* (7), were found in *H. geysericola*; this is consistent with its predicted heterotrophic physiology.

The sequencing of *H. geysericola*, along with *Herpetosiphon aurantiacus* (4), completes the genomic knowledge of the cultured members of the genus *Herpetosiphon*. These data provide important constraints that help ordinate the acquisition of phototrophy, aerobic respiration, and carbon fixation within the *Chloroflexi*.

### Nucleotide sequence accession number.

This whole-genome shotgun project has been deposited in DDBJ/EMBL/GenBank under the accession no. LGKP00000000.
